# High-Efficiency, Near-Diffraction Limited, Dielectric Metasurface Lenses Based on Crystalline Titanium Dioxide at Visible Wavelengths

**DOI:** 10.3390/nano8050288

**Published:** 2018-04-28

**Authors:** Yaoyao Liang, Hongzhan Liu, Faqiang Wang, Hongyun Meng, Jianping Guo, Jinfeng Li, Zhongchao Wei

**Affiliations:** Guangdong Provincial Key Laboratory of Nanophotonic Functional Materials and Devices, School for Information and Optoelectronic Science and Engineering, South China Normal University, Guangzhou 510006, China; lyy777@m.scnu.edu.cn (Y.L.); fqwang@scnu.edu.cn (F.W.); hymeng@scnu.edu.cn (H.M.); guojp@scnu.edu.cn (J.G.); lijinfeng@m.scnu.edu.cn (J.L.)

**Keywords:** metasurface, metalens, phase modulation, integrated devices

## Abstract

Metasurfaces are planar optical elements that hold promise for overcoming the limitations of refractive and conventional diffractive optics. Previous metasurfaces have been limited to transparency windows at infrared wavelengths because of significant optical absorption and loss at visible wavelengths. Here we report a polarization-insensitive, high-contrast transmissive metasurface composed of crystalline titanium dioxide pillars in the form of metalens at the wavelength of 633 nm. The focal spots are as small as 0.54$\lambda_{d}$, which is very close to the optical diffraction limit of 0.5$\lambda_{d}$. The simulation focusing efficiency is up to 88.5%. A rigorous method for metalens design, the phase realization mechanism and the trade-off between high efficiency and small spot size (or large numerical aperture) are discussed. Besides, the metalenses can work well with an imaging point source up to ±15° off axis. The proposed design is relatively systematic and can be applied to various applications such as visible imaging, ranging and sensing systems.

## 1. Introduction

In recent years, manipulating electromagnetic waves has become a vital research topic with the development of integrated optical circuits and optical communication systems. Metasurfaces, composed of arrays of resonators at an interface with subwavelength spatial separation, have attracted a lot of attention due to their excellent performance and ultrathin thickness compared to conventional bulk optical components. By modifying the phase, amplitude and polarization of light waves with extreme freedom, they can arbitrarily control light beams at the subwavelength scale, which makes them suitable for photonic integration and mass production [1,2,3,4]. To date, metasurfaces based on various cells ranging from the V-shaped [1,5,6,7], U-shaped [8], cross-shaped [9] and L-shaped [10] nanoantennas to nanobricks [11,12,13] and Pancharatnam-Berry phase [14] elements [15,16,17,18,19,20] have been proposed to achieve transmissive flat lenses. However, their efficient operation range has seldom worked at the visible wavelength because of the intrinsic optical loss of the constituent materials (usually consisting of either silicon or plasmonic noble metals), which leads to the reported metalenses demonstrated at visible or near infrared wavelengths [5,21,22] often having ordinary efficiencies of below 60%. Besides, realizing full control over phase (2π) of the light often requires precise and high-aspect-ratio nanostructures [12,19,20,23], in which the separation between antennas is facing two conflicting perspectives (Figure S1): On the one hand, to achieve high efficiency, one would prefer to have smaller spacing between antennas, then the phase would be finely sampled. On the other hand, apart from the fabrication problems, there is still a lower limit for the spacing. It is expected that every antenna be electromagnetically independent of its neighbors, so that the phase at the local control points is really implemented locally at the level of every individual antenna rather than over an extended area covering a few neighboring antennas [24]. Indeed the antenna spacing is an essential parameter for the design of metalens, while few works pay attention to it. Additionally, the reported metalenses were usually designed specifically for linear [11,12] or circular [19,20] polarizations of incident light, which have limited their potential application in polarization-independent devices.

In this paper, we propose the use of crystalline titanium dioxide (TiO_2_) as a metasurface material for visible light operation due to its sufficiently high refractive index and especially low loss at visible wavelengths. Through the analysis of the transmission coefficient and the phase by the radii and spacing of varying TiO_2_ pillars, a suitable spacing is chosen. These two points ensure that the proposed metalenses have an efficiency as high as 88.5% and a focusing spot size as small as $0.54\lambda_{d}$ at the design wavelength of 633 nm. Besides, the trade-off between high efficiency and small spot size (or large numerical aperture) along with the focal length and wavelength are discussed respectively in detail. Further investigations prove that the coupling effect between the antennas is negligible and that the phase realization mechanism is mainly due to the waveguiding effect rather than the Fabry-Perot effect for larger radii. Furthermore, the metalenses are polarization-independent, which means they are suitable for both linear and circular polarization, simultaneously. Finally, the metalenses can not only focus the designed plane wave, but also image point sources up to ±15° off axis. We envision that the proposed material can pave the way for the design of many visible metasurfaces, such as metalenses, axicons, beam splitters and so on.

## 2. Methods

**Figure 1:**
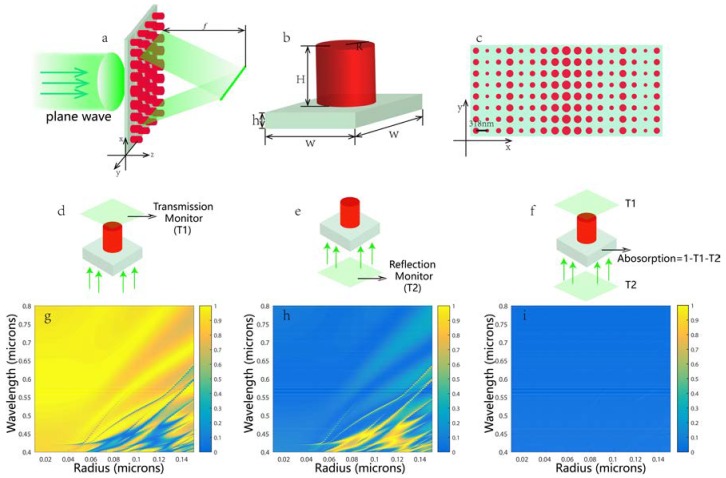
(**a**) The three-dimensional schematic of TiO_2_ antenna arrays with equivalent separation formed on a silica substrate. A plane wave is incident to the antenna array from the silica substrate, and the focus length is *f*. The focus is a narrow line in the y-direction; (**b**) Schematic of the unit cell of the metalens: the circular TiO_2_ post with a height of 488 nm on a 200-nm-thick silica substrate. The size (*W*) of the unit cell is 318 nm × 318 nm; (**c**) Schematic of the metalens from the view of the negative direction of the z-axis; (**d**–**f**) The schematic for recording the transmission, reflection and absorption coefficient of every nanopillar; (**g**–**i**) The according result of transmission, reflection and absorption coefficient map for different nanopillars with the radii ranging from 10–150 nm in the entire visible spectrum.

Figure 1a is the three-dimensional schematic structure: a plane wave is normally incident to an array of circular TiO_2_ posted on a subwavelength silica square lattice, then the transmitted light is focused at a distance of f away from the exit surface of the metalens. The fundamental unit cell of the antenna array is shown in Figure 1b, the size of which is set as *W* in both the x- and y-directions. Figure 1c is schematic of the metalens from the view of negative direction of the z-axis. Here crystalline titanium dioxide was chosen as the component material due to its sufficiently high refractive index and low loss at the visible spectrum. To prove that, we investigated the crystalline titanium oxide’s transmission, reflection, and absorption coefficient on the subwavelength scale for the entire visible wavelength. As shown in Figure 1g–i, we found that most of these nanopillars have high transmittance, low reflectance and almost zero absorption loss in the visible spectrum. The method used to record the data is presented in Figure 1d–f.

To function like a spherical lens, the phase profile φ(x,f) of the metalens needs to follow [5]:

(1) $$\varphi (x,f)=2m\pi +\frac{2\pi }{\lambda_{d}}(f-\sqrt{f^{2}+x^{2}})$$

where *m* is an arbitrary integer number and *λ* is the incident wavelength. Once the required phase discontinuity at a specific center position *x* in Figure 1c is determined, the proper disc radius will be chosen such that its phase response will be closest to the calculated value φ(x,f). There was no phase modulation in the y-direction, resulting in a focal line rather than a point in the focal plane (Figure 1a). The total phase function was realized by subwavelength antennas of fixed center-to-center separation.

**Figure 2:**
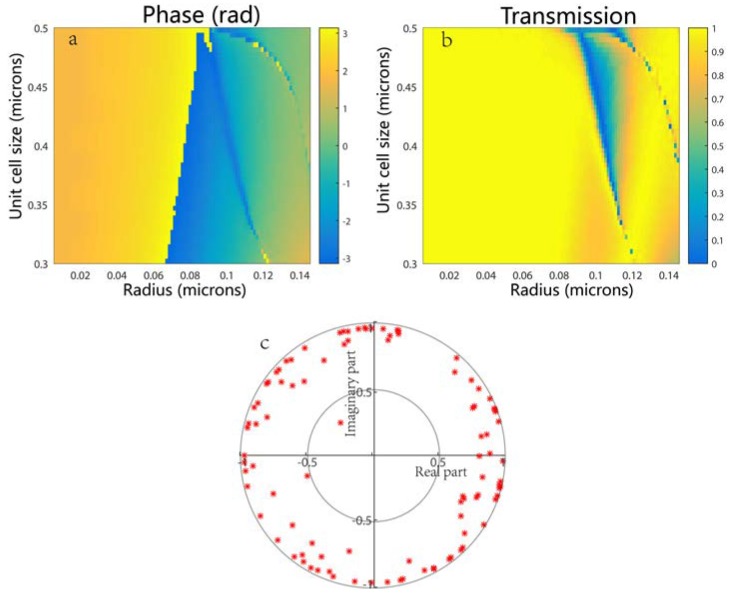
Calculation of (**a**) the transmission and (**b**) the phase of the periodic TiO_2_ posts on a square lattice with different unit cell sizes and radii; (**c**) Complex transmission coefficients for the design wavelength. Each point represents the amplitude and phase of the transmission of a nanopillar with radius *R*.

To illustrate the capability of the proposed ${\mathrm{TiO}}_{2}$ metasurface in transmission and its full 2π phase control, a numerical calculation using the finite time domain difference (FDTD) method was performed and then the phase φ(R) (Figure 2a) and transmission coefficient T(R) (Figure 2b) of period TiO_2_ pillars were analyzed by changing the unit cell size (antenna spacing) from 300 nm to 500 nm and the radius from 10 nm to 150 nm at the wavelength of 633 nm; the resolution of the sampling of the radius and the cell size was 100 × 100. In the calculation, the height of the TiO_2_ post was fixed at *H* = 488 nm, the thickness of the silica substrate (the refractive index *n* = 1.45) is *L* = 200 nm and periodic boundary conditions were applied in the x- and y-directions, and perfectly matched layers were employed along the propagation of incident light (z-axis). As shown in Figure 2a,b, arrays of posts with a *W* = 318 nm unit cell size can smoothly achieve large transmission amplitudes while spanning the full range of phases 2π by varying the radius of the pillars from 10 nm to 150 nm. Figure 2c shows the complex transmission coefficients ($T(R)e^{i\varphi (R)}$) at the design wavelength of 633 nm for a range of radius required to give 2π phase coverage. Each point in the complex plane represents the amplitude (transmission) and phase of a nanopillar with radius *R*, for the given unit cell size *W* = 318 nm and nanopillar height *H* = 488 nm at the design wavelength. High transmission (latitude direction) and close to 2π phase coverage (longitude direction) is evident for the design wavelength.

To gain a better insight into the phase realization mechanism, here we introduced a single step- index circular waveguide model and calculated the phase imparted solely by the waveguiding effect. As shown in Figure 1b, the TiO_2_ nanopillar serves as the fiber core of the refractive index $n_{1}$ and radius *R*, surrounded by the cladding of the refractive index $n_{2}$ (here the cladding is air); the nanopillar height *H* is the propagation length. This phase is given by [25]:

(2) $$\varphi_{WG}=\beta H=\frac{2\pi }{\lambda_{d}}n_{eff}H$$

where β is the propagation constant and $n_{eff}$ is the effective index of the fundamental mode ($HE_{11}$). By varying the diameters of the nanopillars as a function of their position *x*, the effective index of the propagating mode was changed to achieve the desired phase profile (Equation (1)). The $n_{eff}$ can be computed using a single step-index circular waveguide model for $HE_{11}$ modes as in the following [26]:

(3) $$\frac{J_{0}(hR)}{haJ_{1}(hR)}=-(\frac{n_{1}{}^{2}+n_{2}{}^{2}}{2n_{1}{}^{2}})\frac{K_{1}{}^{\prime }(qR)}{qRK_{1}(qR)}+(\frac{1}{{\left(hR\right)}^{2}}-S)$$

(4) $$S=[(\frac{n_{1}{}^{2}-n_{2}{}^{2}}{2n_{1}{}^{2}}{)}^{2}(\frac{K_{1}{}^{\prime }(qR)}{qRK_{1}(qR)}{)}^{2}+(\frac{\beta }{n_{1}k_{0}}{)}^{2}(\frac{1}{{\left(qR\right)}^{2}}+\frac{1}{{\left(hR\right)}^{2}}{)}^{2}{]}^{1/2}$$

In which $n_{1}=2.58,\;n_{2}=1$, $J_{0,1}$ are the first kind of Bessel functions of order 0, 1, $K_{1}$ is the second kind modified Bessel function of order 1 and *h*, *q* are given by:

(5) $$h^{2}={\left(n_{1}k_{0}\right)}^{2}-\beta^{2},\;r<R$$

(6) $$q^{2}=\beta^{2}-{\left(n_{2}k_{0}\right)}^{2},\;r>R$$

**Figure 3:**
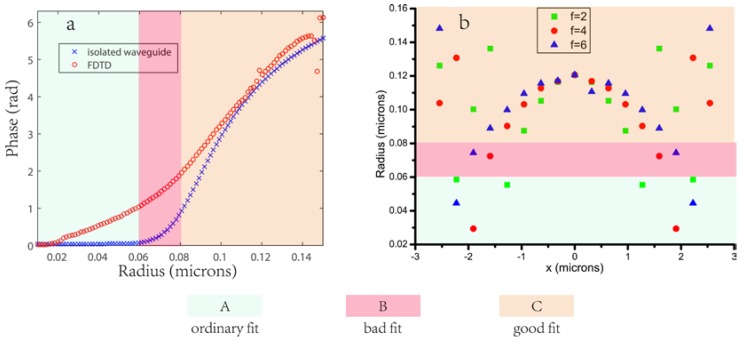
(**a**) Comparison of the phase calculated by finite difference time domain simulation of the building block (nanopillar on a glass substrate) as a function of radius *R*, and the phase due to propagation in an isolated cylindrical waveguide, considering just its fundamental mode $HE_{11}$ at $\lambda_{d}$ = 633 nm. The areas A, B, and C represent different degrees of fitting for two methods; (**b**) The nanopillars selected for the design of metalenses with a focal length of 2, 4, and 6 μm respectively.

**Figure 4:**
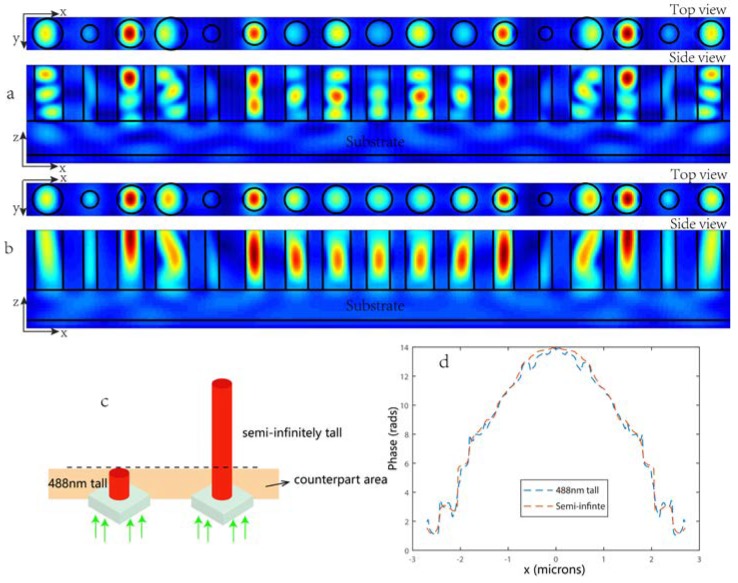
(**a**) Top and side views of the magnetic energy density in the metalens designed for *f* = 2 μm for different post radii *R*; (**b**) Top and side views of the magnetic energy density of the counterpart in semi-infinitely tall TiO_2_ pillars. The black lines depict the boundaries of the TiO_2_ posts. A plane wave with a magnetic energy density of 1 is normally incident on the TiO_2_ posts from the substrate direction; (**c**) The schematic of 488-nm tall and semi-infinite tall nanopillars, respectively. The orange area here represents the counterpart area for comparison. The black dotted line represents the position, 12 nm above the transmitted facet of 488-nm pillar, for recording the phase profile; (**d**) Blue dashed line: Phase distribution recorded at a distance of 12 nm above the transmitted facet of the metalens with the default focal length of 2 μm. The red dashed line: The phase distribution recorded at the same place for the second case in which the TiO_2_ pillars were semi-infinitely long to exclude any Fabry-Perot effects.

Through this model, the relationship between the *R* and β can be directly obtained and then the phase φ(WG) can be calculated by Equation (2). As shown in Figure 3a, the phase calculated by this model follows the one calculated via a finite difference time domain (FDTD) analysis of a nanopillar on a glass substrate with the size of *W* = 318 nm. Here we analyze the fitting degree of the two cures at three intervals represented by areas A, B, and C, with different colors, and investigate the possibility of different areas that will be used for the metalens design. As shown in Figure 3b, most nanopillars’ radii (~80%) are gathered in area C, very few are in area B (~10%). Besides, with the increase of the design focal length, the radii tend more to area C. Thus most nanopillars picked out for the proposed metalenses belong to area C, they are generally suitable for the waveguide model. We envision that the absolute phase difference between the two methods in area B and C is attributed to the mode confinement effect: In these two regions, the fundamental modes for different radii are excited and the reason that two curves fit better for larger radii is that the confinement of the fundamental mode increases. In area A, the fundamental mode has not (or hardly) been excited so that their phases (or $\beta ,n_{eff}$) remain unchanged with the radii increase, which can be proved by the magnetic energy density for small radii seen in Figure 4.

Owing to the high index-contrast, the TiO_2_ waveguides can confine light in a subwavelength region and thus can ideally behave as weakly coupled metasurface phase shift resonators. To demonstrate this, here we performe two simulations for the metalens designed for *f* = 2 μm, in which the TiO_2_ pillars’ height were *H* = 488 nm and semi-infinite, respectively (Figure 4c) [27]. Here we mainly researched the antenna properties that are located at the near field area. Besides, the antenna is 488-nm tall, which makes the counterpart area appear in the near field. Figure 4a shows the top and side views of the magnetic energy density in the metalens for different post radii *R* with a height of *H* = 488 nm. Figure 4b shows the top and side views of the magnetic energy density of the counterpart in the metalens with semi-infinitely tall TiO_2_ pillars. It is apparent for both cases from the top views that the two magnetic energy density distributions are nearly qualitatively identical at the corresponding position and from the side views, so that even as we lengthened the propagation distance, the light was still concentrated inside the posts, thus the electromagnetic coupling effect between posts was negligible. Furthermore, to quantitively prove that the phase realization mechanism is mainly due to the wave guiding effect rather than the Fabry-Perot effect, we also recorded and compared the phase distribution in the same place for the two cases. We know that in an infinitely tall pillar, any Fabry-Perot effects are eliminated and the phase delay is realized by the propagation distance (waveguiding effect). By comparing the counterpart and recording the phase profile in the same place, we can deduce the influence of the Fabry-Perot effect on our 488-nm pillars. As shown in Figure 4d, the blue dashed line represents the phase distribution recorded at a distance of 12 nm above the transmitted facet of the metalens with the pillar height of *H* = 488 nm. The red dashed line represents the phase distribution recorded at the same place for the second case in which the TiO_2_ pillars were semi-infinitely tall. It is notable that the two lines are practically identical, confirming that the Febry-Perot effect did not affect much in the 488-nm tall pillars of our metalens, in other words, the phase accumulation of the pillars is due to the waveguiding effect rather than the Fabry-Perot effect.

## 3. Results and Discussion

### 3.1. The Focal Length Dependence at the Design Wavelength of 633 nm

**Figure 5:**
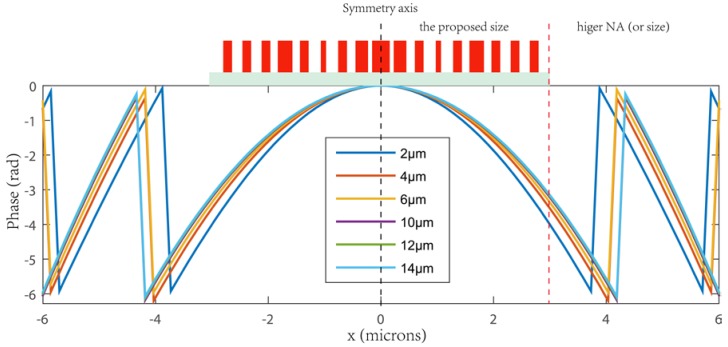
The required phase at each default focal length calculated by Equation (1) in the manuscript, with a known design incident wavelength, $\lambda_{d}$ = 633 nm. The red dotted line implies the size (radius of metalens) of our proposed metalenses. For larger-size or higher-NA metalens designs, one can refer to those phase profiles that go beyond the red dotted line.

**Figure 6:**
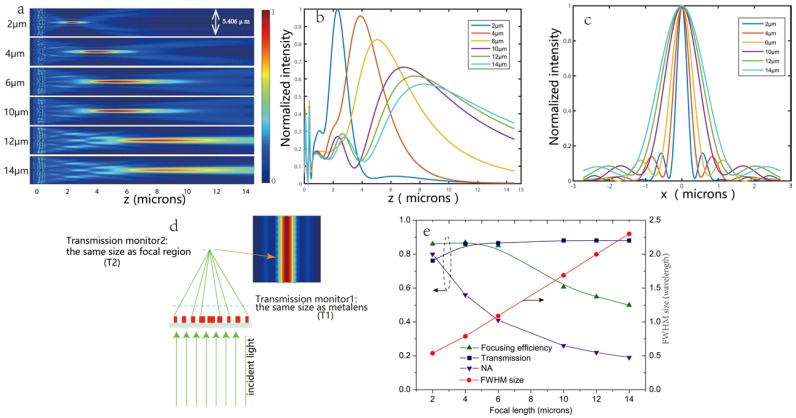
(**a**–**c**) The simulated intensity profiles of the transmitted beam by the metalenses in the xz-plane (**a**) and z axis (**b**) as well as the vertical cut of the focusing spot size at y = 0 (**c**) for several metalenses with f ranging from 2 to 14 μm; (**d**) Schematic of the calculation of the focusing efficiency. The inset picture is the focal plane of the metalens designed for *f* = 6 μm; (**e**) Simulated values of the transmission, focusing efficiency, NA and FWHM spot size for different focal lengths.

The performance of the metalenses was evaluated by three dimension finite difference time domain (FDTD) simulations. We designed a set of 5.4-μm diameter metalenses with a numerical aperture (NA) as high as 0.8 that focused the x-polarized light from the back side of a substrate (200-mm thick fused silica) to points located at distances ranging from *f* = 2 μm to *f* = 14 μm away from the metalenses. We refer to *f* as the focusing length. As shown in Figure 5, the required phase at each default focal length is calculated by Equation (1), knowing the design wavelength, $\lambda_{d}$ = 633 nm, of the metalenses. Our proposed metalenses are all of a size (radius of metalens) within the red dotted line. For larger sizes or higher-NA metalens designs, one can refer to those phase profiles that go beyond the red dotted line. Figure 6a,b show the simulated intensity profiles of the transmitted beam by the metalenses in the x-z plane and z axis with different focal lengths. The two-dimensional focal plane images are shown in Figure S2. Figure 6c is the vertical cut of the focusing spot size at y = 0 for metalenses with different focal lengths. We can clearly see that with the increase in default focal length, the focusing effect became worse, in mainly two aspects: the error between the design value and real value was larger and the focusing spot spread to a bigger size. The simulated values of the transmission, focusing efficiency, numerical aperture (NA) and full width at half maximum (FWHM) spot size for several metalenses with *f* ranging from 2 to 14 μm are presented in Figure 6e. The metalens with *f* = 2 μm has a full width at a half maximum (FWHM) spot size of 344 nm or 0.54$\lambda_{d}$ ($\lambda_{d}$ = 633 nm), which is close to the smallest possible diffraction limited value of 0.5$\lambda_{d}$. A metalens with *f* = 4 μm has the top focusing efficiency of 88.5%, while the largest transmission of 88% was found for metalenses with *f* = 10, 12, 14 μm. In general, higher NAs and smaller spot sizes correlate with higher focusing efficiencies and decreased transmission. The focusing efficiency is calculated as the ratio of the optical power in the focal region to the transmitted incident power, as shown in Figure 6d. The transmitted incident power was defined as the optical power passing through an aperture with the same size as the metalenses at ~λ/2 away from the transmitted facet where the reflection and absorption power are excluded.

In FDTD solutions, we can locate the two positions and extract the transmission through the *T*1 and *T*2 monitor. These transmissions are calculated with:

(7) $$T(\lambda )=\frac{\frac{1}{2}\int \mathrm{real}(\vec{P}{\left(f\right)}^{\mathrm{Monitor}}).d\vec{S}}{\mathrm{sourcepoweer}}$$

This is basically the power flowing through the monitor divided by the source. Thus the focusing efficiency here can be obtained by:

(8) $$\eta =\frac{T2}{T1}$$

### 3.2. The Broadband Characteristics

**Figure 7:**
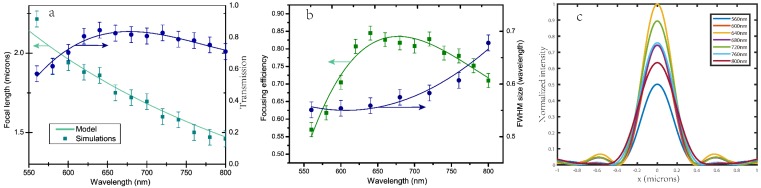
The wavelength dependence of the focal length and transmission (**a**); focusing efficiency and FWHM size (**b**) of the metalens with *f* = 2 μm; (**c**) The vertical cut of the focusing spot for different wavelengths.

The metalenses above were designed to shape the phase fronts for a particular wavelength, however, to characterize the lens performance, it is necessary to research their deviating behaviors for other wavelengths. Here we selected the effective range from 560 nm to 800 nm of the whole visible spectrum, where the metalens can have a transmission and focusing efficiency of more than 50%. Figure 7a shows the wavelength dependence of the real focal length and the transmission of the metalenses with *f* = 2 μm, respectively. The real focal lengths of the simulation results fit well to the diffractive lens according to the formula in the following:

(9) $$f(\lambda )=\frac{f(\lambda_{d})\lambda_{d}}{\lambda }$$

and the transmission of the metalens drop slightly when the wavelength away from the design value of 633 nm. The counterpart of metalens design for *f* = 4 μm and 6 μm is shown in Figure S3a,b, it is clear that the simulation points of the real focal lengths suffer more deviation from the diffractive lens for metalenses with a larger default focal length. Also, the wavelength dependence of the FWHM spot size and focusing efficiency of the metalens with *f* = 2 μm are presented in Figure 7b. We found that the FWHM spot size increased slightly at shorter wavelengths, and the focusing efficiencies remain above 60% for most of the visible range from 560 nm to 800 nm, though it falls from 86% to 51.7%. Figure 7c shows the vertical cut of the focusing spot for different wavelengths. It is intuitive that when moving away from the design wavelength, the intensities become lower and the FWHMs get wider, which indirectly proves the decrease of the focusing efficiency away from the default wavelength.

### 3.3. Insensitive to Incident Polarizations

**Figure 8:**
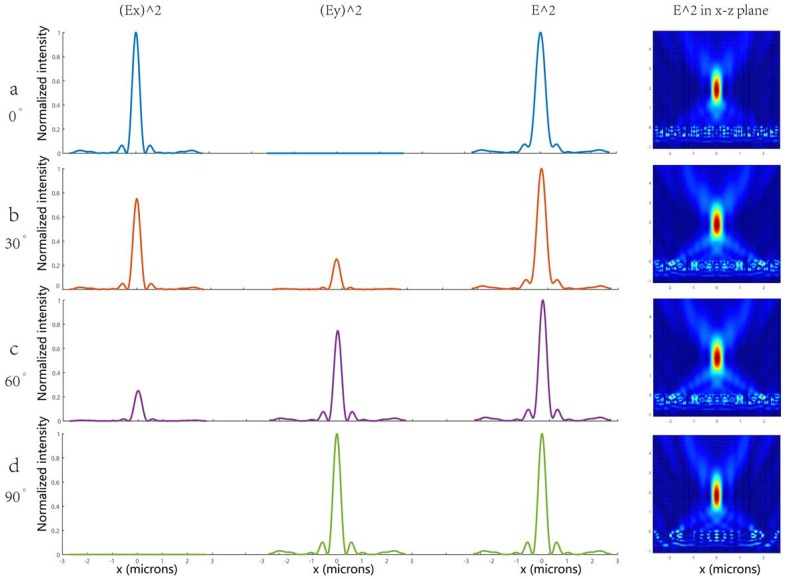
(**a**) The focusing intensities profile at the vertical cuts of focusing spot contributed by x-polarized component and y-polarized component, the total focusing intensities profile and the total intensities distribution in x-z plane for 0° (**a**); 30° (**b**); 60° (**c**) and 90° (**d**) linearly polarized incident lights respectively.

It is worth noting that due to the circular symmetry of the unit cell, the proposed metalenses are polarization-independent. We examined the polarization-insensitivity of the metalens designed at *f* = 2 μm by comparing its focal spot and efficiency for different linearly polarized inputs. Figure 8a–d shows the focusing intensity profile at the vertical cuts of the focusing spot contributed by the x-polarized component and the y-polarized component, the total focusing intensity profile and the total intensity distribution in the x-z plane for 0°, 30°, 60°and 90° linearly polarized incident lights, respectively. As expected, very small changes in the shape and size of the focal spots were observed and the total focusing intensity, which can be regarded as the sum of the x-polarized and y-polarized intensities, remains constant. The standard deviation of the FWHMs of the focal spots for the different polarizations was ~12 nm. In addition, the change in focusing efficiency for different linearly polarized inputs was negligible. As the circularly polarized beam is generated by a superposition of two linearly polarized beams, whose polarization directions are perpendicular and which have phase differences of π/2, the proposed metalenses can also focus on circularly polarized inputs. Here we compared the electric field intensity, electric field and phase distribution of the transmitted light for x-polarized, y-polarizd and circular-polarized incidence lights, respectively, see Figure S4; the matelens performed almost the same focusing phenomenon in each case. Therefore, the designed nanostructure can work very well for both linear and circular polarized incident lights simultaneously, that is to say, unlike previously reported metalenses, the focusing effect of our metalens is insensitive to the incident polarization.

## 4. On- and Off-Axis Imaging

**Figure 9:**
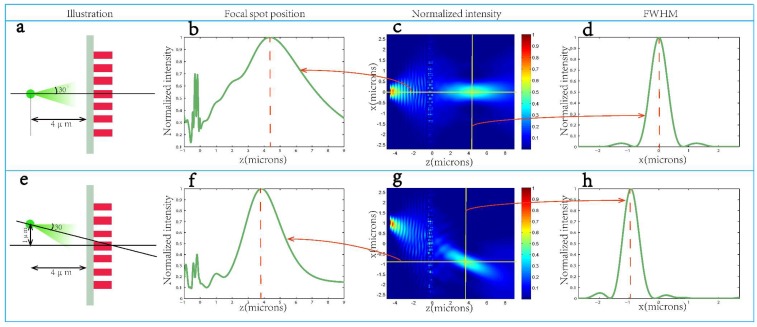
(**a**) Schematic for simulating imaging of an on-axis point source. A point source, modeled as a Gaussian beam with a divergence angle of 30°, is placed 4 μm behind the metalens designed for *f* = 2 μm; (**b**–**d**) The calculated steady electric field intensity |Ex|^2^ of the simulation results on the z-axis (**b**); x-z plane at y = 0 (**c**) and the vertical cut of the focal line (**d**) for the on-axis imaging; (**e**) Schematic for simulating imaging of an off-axis point source. The point source is still 4 μm behind the lens and has a 30° divergence angle, but is now 1 μm off axis to the left of the metalens. The incidence angle toward the center of the lens is approximately 14°; (**f**–**h**) show the intensity distribution, along z direction while 1 μm off the z axis to the right (**f**); on x-z plane at y = 0 (**g**); of the vertical cut of the focal line (**h**).

For imaging purposes, two simulations of imaging on- and off-axis point sources were carried out respectively using a lens with *f* = 2 μm. Figure 9a shows a schematic of the centrally-aligned simulation setup. A point source, modeled as a Gaussian beam with a divergence angle of 30°, was placed 4 μm behind the lens. As the focal length of the lens was 2 μm, an image of the point was expected 4 μm on the other side of the lens according to the Newtonian imaging formula. Figure 9b–d are the calculated steady electric field intensity |Ex|^2^ (The x-component of intensity) of the simulation results on the z-axis, x-z plane and the vertical cut of the focal line. The intensity plot shows a peak near 4 μm on the image side of the lens, close to the expected location. Here we qualitatively show the imaging performance of the metalens by comparing it with a conventional lens. As shown in Figure S5a,b, a conventional lens corrected to focus light from infinity will not perform as well for a nearby object (point source). Since the conventional lens with the same parameter is hard to fabricate, its spherical aberration cannot be specified here. Theoretically speaking, metalenses have no spherical aberration for the design wavelength (Figure S5c), however, due to the scant antennas we used in the metalens and other reasons like the accuracy error of simulation and so on, the metalens we used for the imaging point source still had some aberrations (Figure S5d) of −0.2 μm. Figure 9e shows the off-axis simulation setup; the point source is still 4 μm behind the lens and has a 30° divergence angle, but is now 1 μm off axis to the left. Figure 9f–h are basically similar to Figure 9c,d, respectively. It can be noted that the intensity distribution shows the highest image peak 1 μm off axis to the right and 3.85 μm away from the lens. The image is closer to the lens this time because moving the point source off axis increased its distance from the lens center from 4 μm to 4.12 μm. Besides, for off-axis imaging, it can tolerate ~±15° oblique incidence.

## 5. Conclusions

In summary, we have demonstrated polarization-insensitive, high-contrast transmit-array metalenses composed of crystalline titanium dioxide pillars at the wavelength of 633 nm. The focal spots are as small as 0.54$\lambda_{d}$, which is very close to the optical diffraction limit of 0.5$\lambda_{d}$. The simulation focusing efficiency is up to 88.5%. Further investigation proved that the coupling effect between the antennas was negligible and that the phase realization mechanism of the proposed metasuface was mainly due to the waveguiding effect rather than the Fabry-Perot effect. The focal length’s and the wavelength’s dependence on high efficiency and small FWHM size (or large numerical apertures) was discussed. In addition, the metalenses can work well for an imaging point source within ±15° off axis.

The proposed metalenses work at a specific wavelength, once the metalens is designed (fabricated), the according phase at each antenna is unchanged. We hope to find the method to design a metasurface that is reconfigurable for antenna.

## Supplementary Materials

The following are available online at http://www.mdpi.com/2079-4991/8/5/288/s1.
