# Supplementary material for: High-Efficiency, Near-Diffraction Limited, Dielectric Metasurface Lenses Based on Crystalline Titanium Dioxide at Visible Wavelengths
Source: Nanomaterials (Basel). 2018 Apr 28;8(5):288. doi: 10.3390/nano8050288 (PMC5977302; doi:10.3390/nano8050288)
Supplement: Supplementary file 1 [file nanomaterials-08-00288-s001.pdf]

Supporting Information

High-Efficiency, Near-Diffraction Limited, Dielectric Metasurface Lenses Based on Crystalline Titanium Dioxide at Visible Wavelengths

Yaoyao Liang, Hongzhan Liu *, Faqiang Wang, Hongyun Meng, Jianping Guo, Jinfeng Li and Zhongchao Wei *

1. Critical choice of the nanostructure spacing in waveguide-type metalenses.

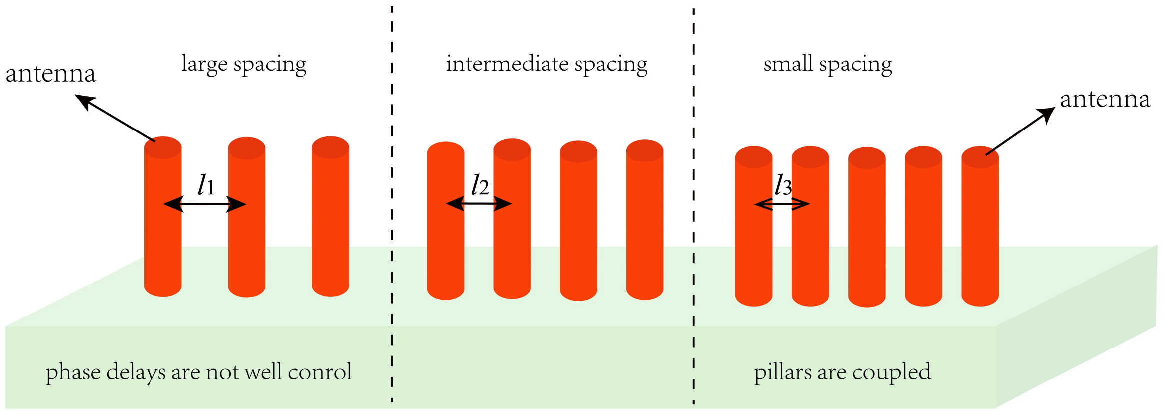

Figure S1. Left panel: nanopillar with large spacings, in which the phase delays are not well control. Center panel: The nanopillars with the suitable spacing, in which pillars are uncoupled, their normalized propagation constant are equal to the effective index of the isolated waveguide. Right panel: nanopillar with small spacing in which the phase sampling is fine but the nanopillars are coupled electromagnetically.

In the manuscript, ”lower limit for spacing” refer to the smallest separation ($l_{\min}$) between the antennas that can be tolerated for waveguide-type metasurface. The separation between antennas is expected to be as small as possible under the condition that no or negligible coupling effect occurred between the neighboring antennas. In this case, the closer sample spacing is, the higher efficiency is. When the spacing comes down to a boundary where the coupling effect cannot ignored, this boundary is regard as “lower limit” or “lower bound”.

We draw the schematic in figure 2 to summarize the main design constrains on the spacing values for waveguide-type metalenses with propagation delays. The left panel corresponding to sample spacings that are two large, the imprinted phase is difficult to accurately contolled. In the right panel, small spacings are considered. The phase sampling is fine while the nanopillars are coupled electromagnetically, which will also lead to the decrease of efficiency. In the center panel, the spacing is suitable: The nanopillars are uncoupled, their normalized propagation constant being equal to the effective of the isolated waveguide. Thus every nanopillar can be electromagnetically inpedendent from its neighbors, so that the phase at the local control points is really implemented locally at the level of every individual nanopillar rather than over an extended area covering a few neighboring nanopillars. These requirements apply to both waveguide-type and resonant metalenses.

2. 2D focal plane images

Two dimensional focal plane images obtained by the simulation at design focal length of 2μm, 4μm, 6μm, 10μm, 12μm and 14μm are shown in Figure S1(a-f), respectively.

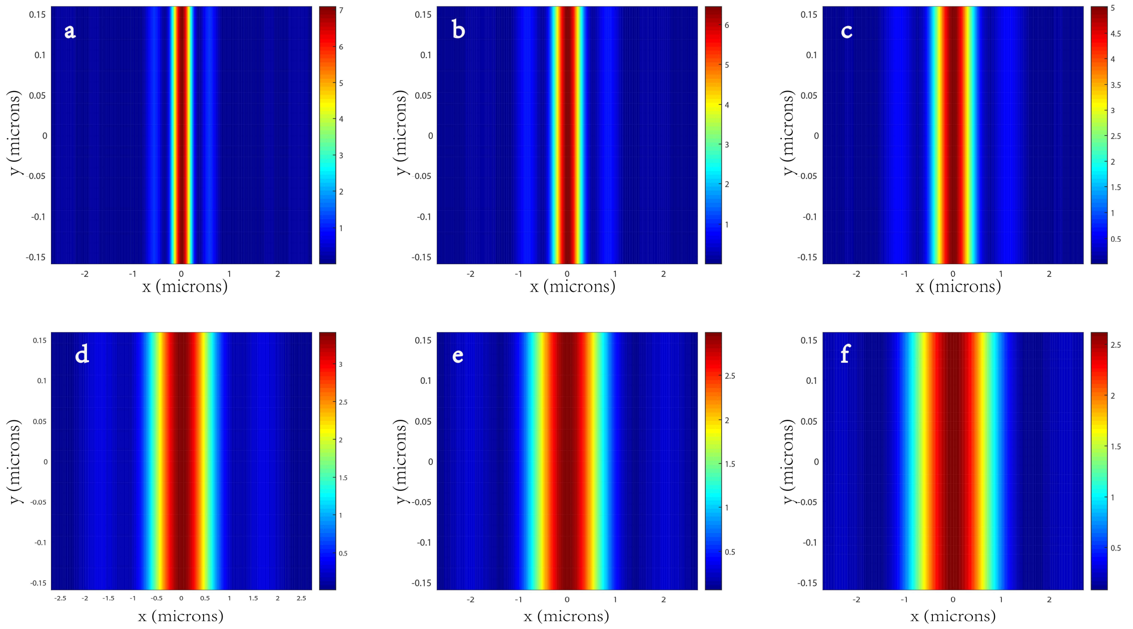

Figure S2 Simulated intensities of focal plane (x-y plane) at (**a**) f=2μm; (**b**) f=4μm; (**c**) f=6μm; (**d**) f=10μm; (**e**) f=12μm and (**f**) f=14μm.

3. The wavelength dependence of metalenses design for f=4μm and f=6μm

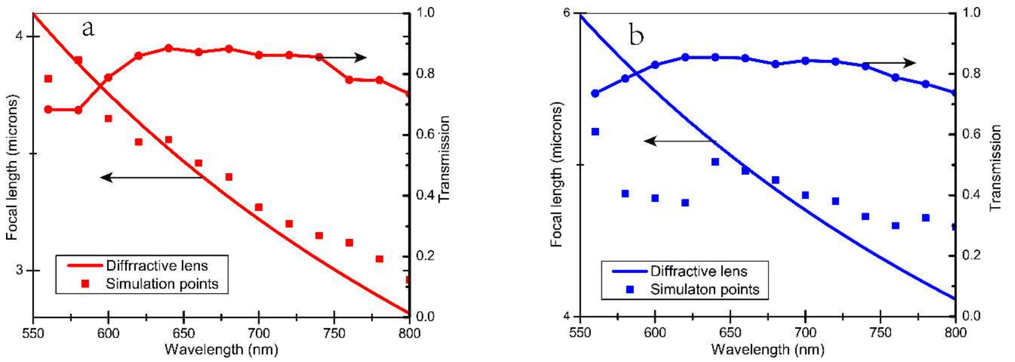

Figure S3 (a-b) The wavelength dependence of focal length and transmission of metalenses design for f=4μm (**a**) and f=6μm (**b**) in the spectrum from 560nm to 800nm.

4. Polarization insensitive to circular-polarized incidence

It has been proved that in the main text that the total focusing intensity, which can be regarded as the sum of x-polarized and y-polarized intensities, remains constant. As the circularly polarized beam is generated by a superposition of two linearly polarized beams, whose polarization directions are perpendicular and phase differences π/2, the proposed metalenses can also focusing circularly polarized inputs. To demonstrate that, the intensities, electric fields and phase distributions with the x-linear polarization(XLP), y-linear polarization(YLP), circular polarization(CP) incidences are

calculated respectively using the metalens designed for f=2μm on the behalf, as shown in Figure S4(a-c), we can easily find that the matelens performs almost the same focusing phenomenon accordingly.

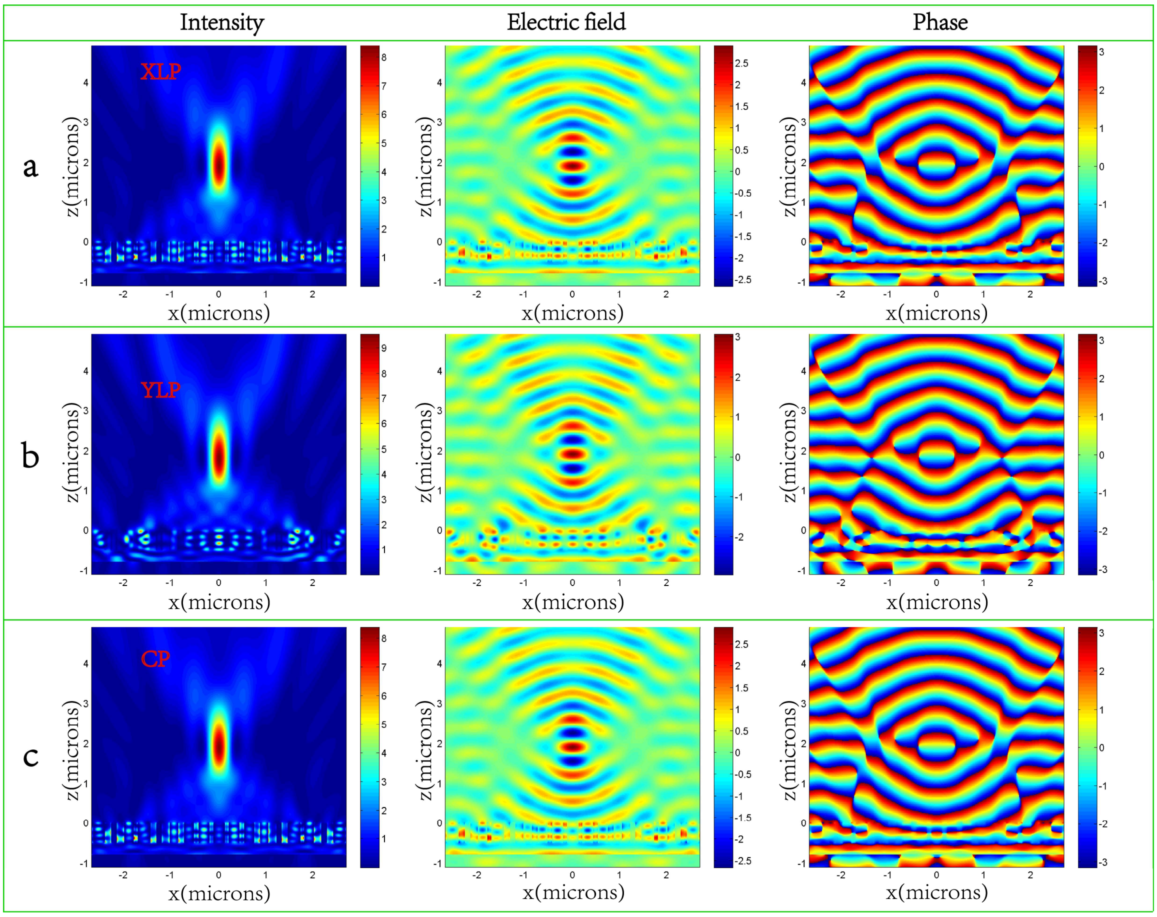

Figure S4 (**a**) The electric field intensities, electric fields and phase distributions of the transmitted light for XLP normal incidence, and (**b-c**) show the similar cases for the YLP, CP incidence lights.

5. The qualitative comparison of the conventional lens and metalens for imaging

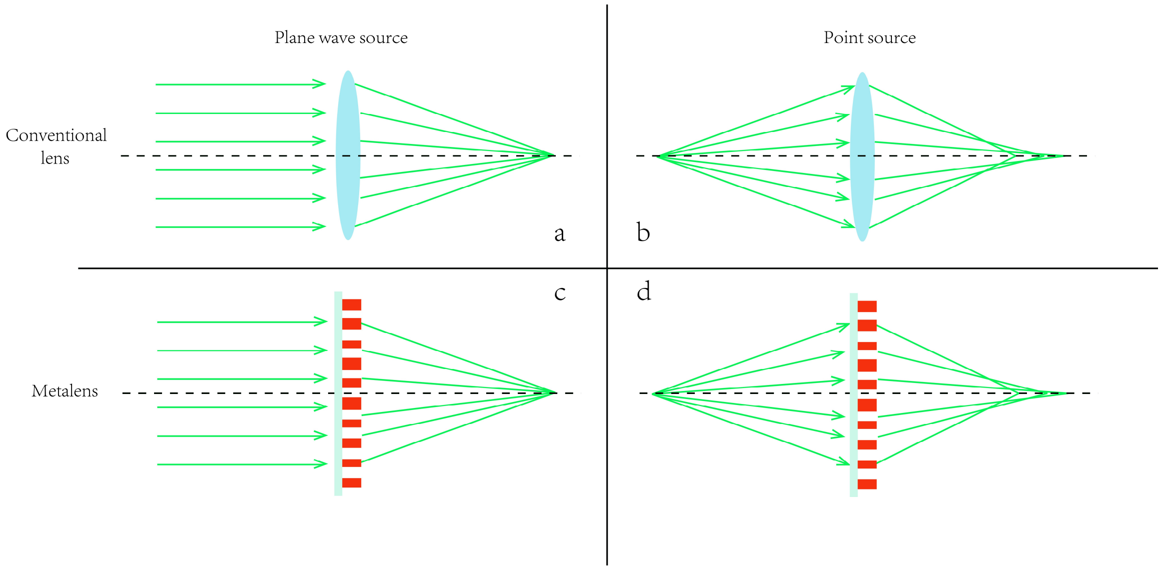

Figure S5. The qualitative comparison of the conventional lens and metalens for imaging plane wave and point source respectively.
